# Central and Peripheral Nervous System Manifestations Associated with Dengue Illness

**DOI:** 10.3390/v16091367

**Published:** 2024-08-28

**Authors:** Maria G. Guzman, Eric Martinez

**Affiliations:** Institute of Tropical Medicine “Pedro Kouri”, WHO/PAHO Collaborating Center for the Study of Dengue and Its Control, Autopista Novia del Mediodia, km 6 1/2, La Lisa 17100, Cuba

**Keywords:** dengue, severe dengue, central nervous system (CNS)

## Abstract

Dengue illness, caused by the dengue viruses, continues to be a major global health concern, with increasing incidence and the emergence of severe manifestations such as neurological complications. An overview of the current understanding of dengue epidemiology, clinical manifestations, and research priorities is presented here. Dengue transmission has escalated in recent years, exacerbated by factors such as vector expansion, climate change, and socioeconomic challenges. The clinical spectrum of dengue ranges from mild febrile illness to severe manifestations, including hemorrhagic fever and neurological complications. Neurological manifestations of dengue, once considered rare, are now increasingly reported, encompassing encephalitis, myelitis, and Guillain–Barré Syndrome, among others. Diagnosis primarily relies on laboratory methods such as RT/PCR, NS1 antigen detection, and serological assays. Despite advancements in understanding the dengue pathogenesis, there remains a critical need for effective vaccines, antiviral drugs, improved surveillance methods, predictive models for disease severity, and long-term studies on post-Dengue sequelae. Integrated programs and holistic approaches to dengue control are essential for mitigating its impact. Addressing these research priorities will be pivotal in combating dengue and reducing its global burden.

## 1. Introduction

According to the World Health Organization (WHO), the global incidence of dengue has increased over the last decades. Between 2000 and 2019, there was a tenfold increase in reported cases worldwide, rising from 500,000 to 5.2 million. In 2019, dengue transmission was reported in 129 countries across the globe [1]. However, during the COVID-19 pandemic, dengue reporting decreased, likely due to diminished surveillance activities. In 2023, a significant increase in cases was observed globally, with simultaneous outbreaks and disease spreading to previously unaffected areas.

**Figure 1:**
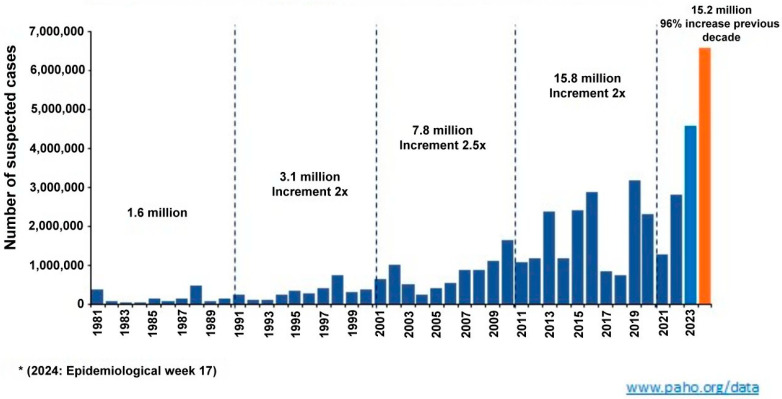
Number of dengue cases, American region, 1981–2024 *.

While dengue transmission was reported in all WHO regions in 2023, the American region experienced the highest number of reported cases, reaching 4.1 million, the highest figure in its history accounting for approximately 80% of the world’s dengue report (Figure 1) [2,3]. It is worth noting that the global report may be underestimated since primary dengue infections are frequently asymptomatic and case reporting is not mandatory in several countries.

Several factors contribute to the increasing transmission of dengue. These include the expansion and increased density of vector populations, primarily *Aedes aegypti* mosquitoes, influenced by climatic variables such as increased rainfall, rising temperatures, and humidity. Weaknesses in surveillance and health systems, human mobility facilitating the spread of the dengue viruses from one area to another, high population density, poverty and inequities also play significant roles in dengue transmission. The economic crisis resulting from the COVID-19 pandemic has further exacerbated these challenges [4,5].

While *Aedes aegypti* is the primary vector for dengue transmission, *Aedes albopictus* also plays a significant role in some Southeast Asia countries and has been detected in parts of Europe, posing risks for dengue and other arboviruses in these regions. In summary, social and climatological factors that promote mosquito breeding sites and mosquito–human contact enhance dengue virus transmission where the viruses are present [6].

The increase in the number of dengue cases has been associated progressively with an increasing report of cases with neurological complications, which is the subject of this report.

## 2. Dengue Virus and Diagnosis

Dengue illness is caused by any of the four dengue serotypes named DENV-1 to 4. DENVs belong to the *Flavivirus* genus together with important human pathogens such as Yellow Fever, West Nile, Zika, Japanese encephalitis B, Tick-borne encephalitis, and St. Louis encephalitis viruses, all belonging to the Flaviviridae family [7]. DENVs are enveloped RNA viruses. The viral RNA codes for seven nonstructural (NS) and three structural proteins, the capsid (C), the membrane (M), and the envelope (E). The C protein forms the nucleocapsid. The prM glycoprotein represents the precursor of the mature M protein and acts as a ‘chaperone’ to aid the folding and maturation of E protein. The E protein is the main target for neutralizing antibodies and it is involved in the protective immunity and the Antibody-Dependent Enhancement phenomenon (ADE). E protein also mediates receptor binding and pH-dependent membrane fusion activity. NS1 is involved in RNA replication and disease pathogenesis; NS3 acts as the viral protease and NS5 is considered the RNA polymerase responsible for viral RNA replication. Molecular epidemiologic approaches have demonstrated the genetic variability of DENVs. Several genotypes in each serotype as well as different lineages in each genotype have been identified [8]. Virus genetic differences may influence the virus replication, viral transmission, and disease severity as well as may be associated with geographical distribution and epidemic potential.

Currently, dengue laboratory diagnosis is mainly performed by genome detection using real-time RT/PCR, NS1 antigen detection (using rapid test or immune-enzymatic platform) during the acute phase of illness, and the IgM serological detection (as an indicator of a recent infection in serum samples collected after day 5–6 of fever onset). Today, the confirmation of DENV infection by serology is complicated in areas with two or more circulating flaviviruses due to the cross-reactivity of the antibody response to cross-reactive epitopes on the flavivirus E protein [9].

## 3. Dengue Epidemiology

The 2024 WHO dengue alert highlights the re-emergence of this illness and the current and future threats it poses to many countries [1]. Previously, Chikungunya fever (caused by the chikungunya virus) at the end of 2013 and Zika fever in 2014–2015 (caused by the Zika virus) were reported in the American region [10]. The epidemic periods of both illnesses represented a new stage in the history of arboviruses [11,12].

Additionally, the current report of the Western Equine Encephalitis outbreak, in Argentina and Uruguay [13], along with the gradually increasing reports of outbreaks of Oropouche (Brazil 2024) and Mayaro [14,15] in the Amazonas, and previous reports of West Nile Fever in the Americas, signify a shift in the epidemiology of these neurological diseases with the threat of their current and future extension.

Transmission of DENVs occurs in a human–mosquito–human cycle [16]. Mosquitoes acquire the virus by feeding on an infected human´s blood. The virus replicates in the mosquito’s midgut for three to five days before moving on to the salivary glands. The virus may then infect a human through the bite of an infected mosquito [17].

## 4. Dengue Clinical Picture

Dengue infection manifests as a systemic and dynamic disease. Following an incubation period of 3 to 10 days, human infection may be asymptomatic but can evolve into dengue with or without warning signs to a severe dengue [18]. Dengue presents as an acute febrile illness accompanied by two or more manifestations such as nausea, vomiting, headache, rash, retroorbital pain, myalgia, arthralgia, petechiae, leukopenia, and a positive tourniquet test (febrile phase of illness). Some cases may exhibit warning signs such as intensive and continuous abdominal pain, persistent vomiting, mucosal bleeding, ascites, pleural or pericardial effusion, lethargy or irritability, and hepatomegaly with progressive hematocrit increase. Severe cases are characterized by plasma leakage, severe hemorrhages, and severe organ involvement leading to death in some patients. Adult chronic diseases, such as hypertension, diabetes, renal insufficiency, and others have been associated with severe dengue [19,20].

Defervescence, occurring around 3–7 days into the illness when the temperature drops to 37.5–38 °C or less, marks either illness recovery or clinical deterioration with warning signs, indicating hypovolemic shock. Most warning signs result from increased vascular permeability and should alert medical doctors to the onset of the critical phase of illness. The period of clinically significant plasma leakage typically lasts 24–48 h. Progressive leukopenia followed by a rapid decrease in platelet count usually precedes plasma leakage, but not always. Patients with increased capillary permeability may deteriorate due to the lost plasma volume leading to shock when a critical volume of plasma is lost. At the beginning, shock is detected by tachycardia, narrowness of pulse pressure, and cold skin. Later, hypotension is evident, being a late sign of shock. Shock is the most frequent cause of dengue severity.

**Figure 2:**
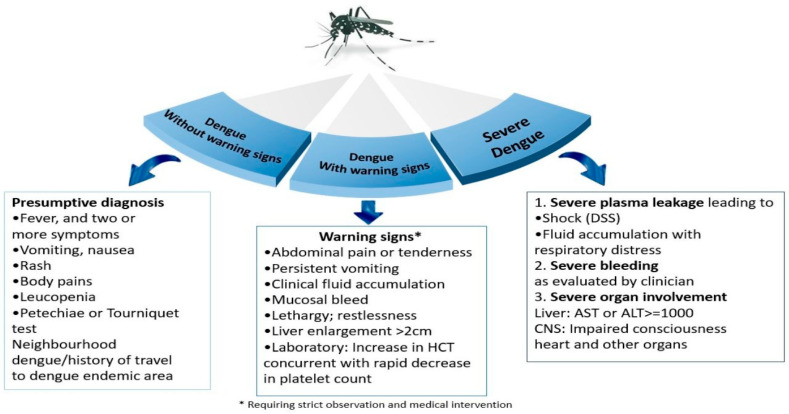
Dengue clinical classification [18].

Organ hypoperfusion from prolonged shock results in hemorrhages, metabolic acidosis, and disseminated intravascular coagulation. Severe hepatitis, encephalitis, and myocarditis and/or severe bleeding can occur with or without plasma leakage or shock [21]. Multiple organ failure may present as a severe complication [22] (Figure 2).

## 5. Neurological Affectation in the Course of Dengue

Neurological manifestations as atypical symptoms of dengue infection were first documented in 1976 [23]. After 1990, there were increasing reports of encephalitis secondary to dengue fever. This led to the evidence of the neurotropic nature of DENVs [24]. Additionally, there were multiple reports of isolation of DENVs from neural tissue and cerebral spinal fluid (CSF) in patients with dengue encephalitis [25]. Of importance is that the clinical manifestations of encephalitis are difficult to differentiate from encephalopathy because both present with reduced level of consciousness and seizures. Actually, in literature, the terms encephalopathy and encephalitis have been used interchangeably.

The global spread of dengue is probably the main factor involved in the incidence increase in cases with neurological complications that has risen from approximately 0.5% to 20% [26]. As DENV infections are becoming increasingly common, consequently, more people may be at risk of experiencing neurological problems. Another important factor associated with the increase in Central Nervous System (CNS) dengue cases is the application of the WHO 2009 classification directed to identifying patients with severe dengue to provide early and intensive care. Among these patients, the classification recognizes as cases of severe dengue those with neurological involvement, in addition to those with hemorrhagic manifestations and circulatory failure [18].

Today, it is now widely acknowledged that dengue can affect the CNS [27,28,29], often indicating severe disease or a risk factor for progression to severe illness [27,30].

The spectrum of neurological complications associated with dengue is broad, encompassing symptoms such as headache, confusion, seizures, hemiparesis, and coma. Both MRI (magnetic resonance imaging) and CT (computed tomography) scans are employed to identify affected areas within the CNS.

Dengue-related CNS involvement typically includes impaired consciousness, neck stiffness, focal neurological signs or seizures. The diagnosis of *Dengue encephalopathy* is considered if these symptoms occur alongside hepatic failure, metabolic acidosis, severe hyponatremia, prolonged shock, disseminated intravascular coagulation, or brain hemorrhage with normal Cerebral Spinal Fluid (CSF) in confirmed dengue patients. *Dengue encephalitis* is characterized by dengue CNS involvement and the presence of DENV RNA, IgM, or NS1 antigen in CSF.

Various neurological manifestations have been observed including myelitis, encephalitis, myositis, and Guillain–Barré Syndrome (GBS) [31], as well as diverse neuromuscular complications associated with dengue [32,33,34,35]. Intrathecal synthesis of immunoglobulins (IgA, IgG, and IgM) has been identified as a contribution to the diagnosis of CNS diseases including dengue [36,37].

The reports of neurological complications span different regions. In Brazil, cases of meningitis accompanied by symptoms such as headache, fever, vomiting, and neck stiffness/rigidity have been documented in dengue cases [38]. In Taiwan, hemorrhagic stroke with symptoms including dysarthria, headache, vomiting, hemiparesis, and somnolence has been reported [17]. In Guadeloupe, neurological complications of dengue including sphincter disturbances, abrupt motor and sensory neurons, and spinal lesions at three vertebral segments have been observed [38]. In Sri Lanka, unusual and severe manifestations such as encephalitis, encephalopathy, Guillain–Barré syndrome, as well as liver failure, kidney failure, myocarditis, and multi-organ failure were noted in 44 dengue patients with 11 deaths [39]. In Vietnam, several studies associated dengue with neurological complications such as encephalitis, encephalopathy, transverse myelitis, and hemiplegia [24,40,41].

Neurological complications and fatalities have been reported both in children and adults. Affected subjects range from the very young (3 months of age) to older individuals (60 years or older) [29,40,42].

[43] reported dengue neurological complications in children with confirmed DENV infection. In Ceará, Brazil, a dengue epidemic was associated with 80 fatalities aged 3 months to 86 years old with confirmed diagnosis (DENV 2 and DENV 3), showing the following neurologic diagnosis: 46.3% encephalitis, 34.1% meningoencephalitis, and 19.5% meningitis. The major clinical manifestations observed in these individuals were fever, headache, mental irritability, breathlessness, vomiting, muscle pain, tiredness, abdominal pain, somnolence, restlessness, dizziness, cough, seizure, coma, and neck stiffness [44]. Some years ago, Pancharoen et al. (mentioned by Araujo) had described this mainly in children, and later, other researchers have made contributions [24,45,46]. At present, age and probably race are not main items to be considered as determinants although some authors consider young age as a risk factor for neurological complications.

In India, the clinical profile of dengue is evolving [47], with atypical manifestations such as encephalitis with acute intracerebral infarction and facial palsy [48] as well as acute intracranial hemorrhage [49]. Other cases have been associated with acute motor quadriparesis due to hypokalemia [50] and acute encephalitis with gastric hemorrhage [51].

While acute disseminated encephalomyelitis was once considered rare [52], it is now recognized as a relatively common complication of dengue [53,54] including in pediatric ages [55].

Memory loss along with symptoms such as myalgia, weakness, hair loss, reduced resistance to physical effort, headache, reasoning problems, arthralgia, sleepiness, and emotional liability have been observed in dengue patients. These symptoms can persist for more than 14 days with some lasting for 6 months or more [56]. It has been proposed that chronic DENV infection may persist in the CNS potentially contributing to progressive dementia in affected patients [57]. Dengue panencephalitis in a patient with progressive dementia with extrapyramidal features has been described [57], along with the risk of transverse myelitis following dengue infection [58].

Various classifications of neurological complications in dengue cases have been proposed. Carol-Artal et al. categorized them into dengue encephalopathy, encephalitis, immune-mediated syndromes, dengue muscle dysfunction, and neuro-ophthalmic disorders [25]. Others classified them based on metabolic disturbances such as encephalopathy, direct viral invasion as encephalitis, meningitis, myositis, and myelitis and those related to an autoimmune reaction including conditions such as Acute Disseminated Encephalomyelitis (ADEM), optic neuritis, myelitis, and GBS [59,60]. Jugpal et al. [61] considered the spectrum of findings on MRI in the brains of patients with neurological manifestations of dengue fever and Solbrig and Perng categorized them into CNS and eye-related complications, peripheral nervous system syndromes, and immune-mediated syndromes occurring during the convalescent or post-dengue phases [62].

In a recent study, Trivedi and Chakravarty recognized dengue encephalopathy, dengue encephalitis, ocular manifestations, dengue-associated stroke (both ischemic and hemorrhagic), posterior reversible encephalopathy syndrome, immune-mediated neurological syndromes such as mononeuropathies, GBS, acute transverse myelitis, acute disseminated encephalomyelitis, neuromuscular complications such as dengue-associated hypokalemic paralysis, myositis, rhabdomyolysis and myalgias and cerebellar syndromes in dengue [27].

Table 1 summarizes some neurological complications associated with dengue according to [6].

### Dengue Ophthalmic Affectation

Ocular manifestations are increasingly recognized as part of the spectrum of symptoms in dengue patients. Reported ocular complications include subconjunctival hemorrhages, conjunctivitis, anterior and posterior uveitis (including vitritis, chorioretinitis, and retinal vasculitis), maculopathy, retinal hemorrhages, and optic neuritis. While these manifestations typically resolve spontaneously, they can lead to irreversible visual impairment [69]. Additional ophthalmic complications observed during dengue infection include myopic shift, corneal pathology, maculopathy, retinal vein occlusions, posterior uveitis, macular edema, and neuro-ophthalmic manifestations. Common symptoms associated with these complications include metamorphopsia, scotomata, floaters, and blurring of vision [70].

Cases of dengue-associated ocular complications, such as corneal pathology, have been diagnosed in various regions including the Americas, Western Pacific, and Southeast Asia presenting symptoms such as lower corneal erosions and peripheral hypopyon corneal ulcer [71,72]. Retinal vein constrictions have been reported in Malaysia [73] while neuro-ophthalmic manifestations such as diplopia and acute vomiting were documented in India [74]. Other complications like posterior uveitis, macular edema, myopic shift [75], ischemic foveolitis, and outer maculopathy have been reported worldwide [76]. Diagnostic modalities such as tomography, optical coherence tomography, angiography, microperimetry, and near-infrared imaging play important roles in identifying and assessing these ocular situations [77]. Severe complications like retinal hemorrhage, retinal vasculitis, and pan-ophthalmitis can pose a threat to vision and necessitate urgent ophthalmic evaluation [78].

## 6. Dengue Laboratory Observations in Cases with Neurological Complications

Laboratory observations in cases of dengue with neurological complications have provided valuable insights into the pathogenesis of these conditions. DENVs have been isolated and viral antigens have been detected in the human CNS [79]. Moreover, viral RNA has been identified in CSF and intrathecal synthesis of specific dengue antibodies has been reported [47,80]. The neurotropic nature of Dengue Fever (DF) has been confirmed through epidemiological studies, case series and histopathological examinations, with a higher prevalence observed in teenagers and children [38,81].

During an epidemic in Salvador, Ceará, Brazil, among 150 necropsies of studied patients, 84 were dengue positive based on various tests including RT/PCR, viral isolation, IgM, IgG, NS1, and IHC (immunohistochemistry). According to Araujo et al., most of these cases exhibited histopathological evidence for meningitis and/or encephalitis along with cerebral congestion, brain edema, hemorrhage, or brain necrosis (some with multiple findings) [44]. Another study conducted on fatal dengue cases in Cuba demonstrated the presence of apoptosis signals in brain cortex sections, providing further support for its involvement in severe dengue [82]. Studies on human and murine neuronal cell lines inoculated with DENVs have shown that virus replication induces apoptosis [31] suggesting a potential mechanism underlying severe dengue pathogenesis.

## 7. Dengue Pathogenesis and Neurological Disorders

Individuals of all ages exposed to infected mosquitoes are susceptible to DENV infection. Infected female *Aedes* mosquitoes transmit DENVs to humans primarily through mosquito bites. Although humans are not capable of transmitting the virus, it can be transmitted via blood transfusion from an infected to a non-infected person. Once in the bloodstream, DENV can systematically enter and sequentially damage lymph nodes and blood vessels inducing viremia [83].

DENVs utilize membrane receptors and attachment factors on the cell plasma membrane to gain entry into the cytoplasm. The mature virion attaches directly to cellular membrane receptors or uses several attachment factors to initiate the clathrin-dependent endocytic pathway. Within endocytic vesicles, acidification triggers conformational changes in the E protein dimers, leading to the formation of fusogenic trimmers. Ultimately, pores are formed allowing the release of the virus genome into the cytoplasm [84].

Increased levels of various cytokines and chemokines such as interferon-gamma (IFN-γ), granulocyte macrophage colony-stimulating factor (GM-CSF), interleukin-10 (IL-10), macrophage inflammatory protein-1 beta (MIP-1β), interleukin-1 beta (IL-1β), interleukin-8 (IL-8), tumor necrosis factor alpha (TNF-α), IFN-γ inducible protein 10 (IP-10), and monocyte chemoattractant protein-1 (MCP-1) have been associated with the progression to severe dengue [85]. These cytokines are mainly secreted by monocytes.

Virus-specific antibodies [86] produced by plasmablasts form immune complexes, leading to activation of complement and releasing vasoactive anaphylatoxins [87].

Some attribute the severity of dengue to specific virulence factors of certain virus strains. However, Halstead suggests that ADE best explains the correlation of vascular permeability syndrome with a second heterotypic DENV infection as well as the infection in the presence of passively acquired antibodies. NS1, according to growing in vivo and in vitro evidence, is responsible for most of the pathophysiological features of severe dengue including its contribution to capillary leak through activation of the complement system in the presence of antibodies and the activation of platelets that adhere to endothelial cells [88]. Dengue infection can trigger uncontrolled activation of inflammatory cells and an exaggerated release of cytokines, leading to hemophagocytic lymphohistiocytosis [89,90].

Infants born to dengue-immune mothers and individuals with a secondary DENV infection are most susceptible to severe dengue. Infection by any of the four serotypes can cause dengue illness with severe dengue being rare. While primary infection provides life-long protective immunity to the infecting virus, there is only short-lived cross-immunity to the others. Previous infection with a different serotype increases the risk of severe disease due to ADE. In ADE, heterotypic non-neutralizing antibodies form complexes with DENV, enhancing infection of mononuclear phagocytes with a higher number of infected host cells, improving viral replication and worsening clinical signs. Consequently, this may contribute to neurological and other complications.

Katzelnick et al. confirmed that the host’s immune responses can worsen disease. Studies have shown that ADE occurs within a specific range of antibody concentrations. Low levels of antibody do not enhance disease severity, intermediate levels exacerbate the disease, and high antibody titers protect against severe disease [91].

After analyzing the Cuban experience with dengue epidemics, Kouri et al. early proposed an integral hypothesis, stating that specific viral factors interacting with particular host factors (such as age, preexisting antibodies, ethnicity, some co-morbidities and host genetics) in a particular ecosystem are necessary for a severe epidemic [92]. This comprehensive view of the complex phenomenon is now widely accepted, with heterotypic secondary infection being the main risk factor for severity. The time interval between primary and the secondary infection as well as the sequence of the infecting dengue virus serotypes, such as DENV 1-2, DENV 1-3 are also important [93,94,95].

In this complex scenario, some authors emphasize the significance of understanding vector–host–pathogen pathways, and well as the ecology and biology of the Aedes species in connection with DENVs.

Previously considered an exception, DENV neurotropism in humans is increasingly supported by evidence indicating direct neurovirulence of the virus. The mechanisms of DENV neuroinvasiveness, neurotropism, and neurovirulence require further clarification. The hematological route is likely used by the virus to enter the CNS either as a free particle or within an infected cell. The overexpression of cytokines altering endothelial permeability as well as the possibility of the virus crossing endothelial cells through transcytosis (similar to West Nile Virus) are potential mechanisms of DENV neuroinvasiveness [96,97]. Animal studies have shown that the virus is known to release cytokines that could breach the blood–brain barrier, thus being capable of CNS invasion [98]. Detection of the virus or viral antigens, including NS1 detection in the CNS, as well as anti IgM antibodies in the CSF, provides evidence of DENV neurotropism [29,99]. Although neurovirulence and neurotropism are not synonymous, studies in animals and humans suggest the neurovirulence capacity of DENV. Apoptotic cell death observed in the mouse model and human fatal cases supports DENV neurovirulence [100,101].

Dengue encephalitis, an uncommon but serious side effect of DENVs infection, results from the virus directly invading CNS [48]. The pathophysiology of dengue encephalitis is multifaceted and encompasses various mechanisms, including immune-mediated consequences, systemic issues, and direct invasion [102].

Three different pathogenic mechanisms could explain the neurological complications of dengue:

(1) The virus directly invades the CNS, which leads to myelitis, meningitis, and encephalitis;
(2) A systemic condition that brings stroke and encephalopathy;
(3) The para-infection or post-infection immune-mediated conditions such as optic neuritis, acute disseminated encephalomyelitis, and GBS [80].

The presence of virus and/or antibody in the CSF are evidence for dengue neurotropism. It is possible that the virus leaks into the CSF through damaged cerebral vascular endothelium. However, the correlation of virus in the CSF with otherwise unexplainable encephalopathy would point strongly toward CNS infection. Some patients have virus in the CSF but not in the serum [45], suggesting some other mechanism besides the passive viral leak into the CSF during viremia. The detection of viral RNA [79] and antigens in CNS biopsies directly confirm viral infiltration of brain parenchyma. More studies are needed to elucidate the mechanisms by which DENV may penetrate the blood–brain barrier. Some studies support the viral entry through infected macrophages [29] and histamine release [98].

The complex pathophysiology of dengue encephalitis includes host defense mechanisms, viral virulence factors, and the role of mosquito vectors in virus transmission. Japanese Encephalitis (JE), another arboviral disease, and a top cause of viral encephalitis in Asia, is caused by the JE virus, a flavivirus mostly spread by mosquitoes with a complex transmission that involves vectors, hosts, and environmental variables [102].

Viral and host factors can be involved in dengue encephalitis. DENV2 and 3 have been frequently associated with dengue neurological disorders as well as DENV 4 [24,103]. Bordignon et al. identified a DENV 1 neurovirulent phenotype that may provoke meningitis with some amino acid substitutions in the envelope and NS3 proteins [104]. On the other hand, Minh Huong Phu et al. isolated a DENV 3 genotype-III strain from a CSF sample of a DENV encephalitis patient during an epidemic in Vietnam in 2013. This virus showed an enhanced growth in human neuronal cells [105]. The full-length genome sequence demonstrated that a distinct amino acid substitution in the NS2A protein was unique to the CSF-derived DENV 3 strain suggesting that NS2A may be a crucial region in the neuropathogenesis of DENV 3 [105]. Although age (young adults and children) and obesity have been reported as possible host risk factors, more studies are needed to identify host and viral factors.

In Taiwan, a large population-based cohort study has shown that dengue has been associated with an increased short-term risk of a rare complication, autoimmune encephalomyelitis, but not associated with other autoimmune diseases [47].

Table 2 shows some dengue neurological complications associated with direct viral infection of CNS, autoimmune reaction, and metabolic and hemorrhagic disturbances adapted from [106]. Table 3 describes some encephalitis case studies in different countries.

## 8. Discussion

DENVs infection is transmitted through bites of *Aedes* mosquitos, hugely spread in tropical and subtropical environments, mostly found in urban and semi-urban areas. Unfortunately, there is not a particular therapeutic approach, but prevention, adequate awareness, detection at an earlier stage of viral infection, and appropriate medical care can diminish the fatality rates. Substantial advances in our understanding of the DENVs, host immune response, disease pathogenesis, and disease progression have been made within the past decade.

DENV was considered initially as a non-neurotropic virus; however, an early report supports the association of the infection with neurological disorders [26]. The increasing of global dengue transmission has been accompanied by the frequent report of cases with neurological disorders. Currently, the CNS impairment is recognized by WHO as one of the criteria for severe dengue [18]. Dengue-associated neurological complications result in a prolonged course of disease, being reported both in children and adults and associated with the four dengue serotypes.

The neuropathogenesis of DENV infection is still poorly understood. Viral and host factors may play an important role in the neurological disorders associated with dengue. In this context, direct viral infection of CNS, autoimmune reaction, metabolic and hemorrhagic disturbances can be involved in the pathogenesis [122].

Neurological complications in dengue infection become increasingly important as the dengue epidemics continue to occur. The clinical manifestations are diverse and result from different neuropathogenic mechanisms still poorly understood. Novel interventions have emerged as partially effective vaccines and innovative mosquito control strategies. A reliable immune correlate of protection remains a challenge for the assessment of vaccines.

## 9. Conclusions

The detection of DENV and its antigen in the CSF of patients presenting neurological manifestations underscores the direct involvement of the virus in the CNS pathology. This highlights the importance of understanding the intricate mechanism between vectors, hosts, and pathogens in neurological dengue as well as the complex pathophysiological mechanisms involved. With the global increase in dengue cases, it is anticipated that instances of neurological complications will also rise. Therefore, there is an urgent need for in-depth research into the underlying causes and mechanisms of these complications. Comprehensive studies in this area are essential for advancing our understanding of dengue-related neurological disorders and informing clinical management strategies.

## 10. Future Directions (Research Priorities)

- Vaccine Development. Despite efforts, the development of safe and effective dengue vaccines remains a priority. Continued research into vaccine candidates and clinical trials is essential to ultimately prevent DENV transmission [123].
- Antiviral Drug Development. Research efforts should also focus on developing antiviral drugs to treat dengue infection. This includes identifying potential drug targets and conducting preclinical and clinical studies to assess their efficacy and safety [124].
- Improved Surveillance Methods. Enhancing surveillance methods for dengue is crucial for early detection and response to outbreaks. This involves developing more sensitive case identification and laboratory diagnostic techniques.
- Predictive models for Disease Severity. Developing predictive models to identify patients at risk of developing severe dengue and mortality is essential for improving patient care and outcomes. This requires comprehensive research into clinical and genetic risk factors [125,126,127,128,129].
- Long-term Effects and Sequelae. Research should investigate the long-term effects and sequelae of dengue infection, including neurological manifestations and prolonged dengue cases. Understanding these effects will inform clinical management and patient care strategies [130].
- Genetic Studies. Genetic studies on both the DENV and the host are promising avenues for identifying genetic markers associated with disease susceptibility and severity. This includes analyzing gene expression datasets to identify predictive gene sets for severe dengue [131].
- Artificial Intelligence in Surveillance. Evaluating the effectiveness of artificial intelligence-mediated systems in dengue surveillance can improve the quality of surveillance data and aid in early detection and prediction of outbreaks [132].
- Integrated Programs and Holistic Approaches. Implementing integrated programs at national and international levels that take a holistic approach to dengue control is essential. This includes incorporating mosquito control strategies and engaging various stakeholders in dengue prevention and control efforts [133]. By prioritizing these research areas and fostering collaboration between researchers, policymakers, and health care professionals, significant advances in combatting dengue and reducing its global burden can be made.

## Figures and Tables

**Table 1:** Neurological complications associated with dengue infection *.

Complication	Signs and Symptoms and MRI/CT Scan
Cerebellar syndrome	Gait ataxia, dysarthria, horizontal, vertical nystagmus. Brain infractions involving medulla regions and pons regions [38].
Encephalitis	Cerebral involvement indications, seizure with severe headache. Hyperintensities in bilateral cerebral hemispheres including basal ganglia in MRI [63].
Meningitis	Headache, fever, vomiting, neck stiffness/rigidity. Leptomeningeal enhancement and distention of the subarachnoid space in MRI [64].
Disseminated encephalomyelitis	Monophasic course, involvement of multifocal white matter, inflammatory. Demyelinating. Abnormalities in the CNS white matter, with or without gray matter involvement in MRI [65].
Hemorrhagic stroke	Dysarthria, headache, vomiting, hemiparesis, somnolence. Hyperdensity at CT [66,67].
Transverse myelitis	Sphincter disturbances, abrupt of motor, sensory neurons, spinal lesions at three vertebral segments. Medullary lesions at the thoracic and cervical levels [58,68].
Ischemia stroke	Dysarthria, hemiparesis. Acute infarct in right parietal region in MRI [66].

* Adapted from [6].

**Table 2:** Dengue neurological complications according to pathogenic mechanisms.

	Evidence of DENV Neurotropism *	References
**Central nervous system DENV infection**	Encephalitis (seizures, altered consciousness, and headache are the most frequent symptoms). Confirmation in cases with fever, acute signs of cerebral involvement, and a positive laboratory result (anti-dengue IgM or dengue RNA in the serum and/or CSF). Myelitis (uncommon, appears 7–30 days after the onset of illness. Some symptoms may persist, such as paraparesis and sphincter dysfunction). Meningitis (rare, more frequent in children and similar to other viral meningitis).	[106] [107] [108] [109]
**Autoimmune reaction in DENV infection**	Guillain–Barré (GBS) (clinical presentation is similar to other infections, with ascending paraparesis as the main manifestation). Miller Fisher syndrome Neuromyelitis optica Optic neuritis Acute disseminated encephalomyelitis (ADEM)	[110] [111] [112] [113]
**DENV and metabolic disturbance**	Observed during dengue shock syndrome by encephalopathy (brain edema, cerebral anoxia, metabolic acidosis, electrolyte disturbances, vasculitis, liver and kidney failure).	[113]
**Hemorrhagic disturbances in DENV infection**	Observed in acute dengue cases with brain hemorrhage with edema, vascular congestion, and focal hemorrhages.	[114]

* Adapted from [106].

**Table 3:** Dengue encephalitis study summary in different countries *.

Study and Results Summary	Geographical Area/Country	Ages	Laboratory Studies	References
Studied 175 hospitalized patients (159 DF, 12 DHF, 4 DSS), 2011–2013. 115 with unusual manifestations; 13 (7.4%) with encephalitis (10 DF, 2 DHF, 1 DSS). Three fatalities. DENV 2 and 3 were detected.	Asia, South India	18–80 years	NS1, IgM ELISA, RT/PCR.	[115]
One-year prospective study (2004) of 194 children with suspected acute encephalitis. Total of 9 (4.6%) cases with dengue confirmed infection (7 with IgM and 3 with RT/PCR positives at CSF). Mean age, 8 years. One fatal case and one with severe sequelae.	Asia, Vietnam	<16 years	IgM ELISA, RT/PCR	[41]
Specimens and clinical data from cases with suspected viral CNS infections, 1994–2004, were reviewed for dengue infection. Dengue infection was confirmed in 54/401 (13.5%) cases of suspected viral CNS infection. Mean age 9.6 years. Total of 53 serologically confirmed cases, one DENV 2 isolate; 28 (51.8%) with encephalitis, 18 (33.3%) meningitis, 6 (11.1%), seizures (one with encephalitis and one with paralysis), two acute flaccic paralysis/Guillain–Barré syndrome 2 (3.7%). Two fatalities (3.7%).	Americas, Jamaica	8 months–49 years	Viral isolation, IgM ELISA, HI study	[116]
From 5400 hospitalized patients with DHF (1997–1999), of whom 224 were referred to intensive care unit. From them, 27 patients with dengue encephalopathy were enrolled in the study (incidence 0.5% of all patients with DHF). Median age 7 years including 6 < 1 year. Total of 26 patients were in coma, 21 developed generalized convulsions and 1 hemiplegia. Total of 22 with dengue IgM in CSF, one with positive RT/PCR (no serotype detection was reported).	Asia, Vietnam	<16 years	IgM ELISA, RT/PCR	[40]
Studied 1559 patients with acute febrile illness (epidemic of 2002). Total of 831 (53.3%) cases including 40 fatalities were confirmed as dengue infection. DENV 3 was obtained in 99% of cases. Neurologic signs were observed in 1.3% of confirmed patients. One patient with encephalitis, confirmed by DENV 3 detection in CSF.	Americas, Brazil	1–73 y	Viral isolation, IgM/IgG ELISA, RT/PCR Immunohistochemical studies	[117]
Study of 526 hospitalized patients with acute encephalitis syndrome, 2011–2012. Viral aetiology was noted in 91 (17.2%) patients. Dengue was identified in 3 (0.57%) patients. IgM was detected in CSF (1), blood (1); RT/PCR positive in blood (1). One fatality.	Asia, India	<15 y	NS1, IgM ELISA, RT/PCR	[118]
Study of 540 dengue patients, mean age of 37.94 ± 22.027. Total of 27 (5%) with encephalitis, 63% of them ≤20 y. Dengue was confirmed in 17 CSF samples (3 NS1 Ag and 14 IgM detection). Remaining 10 CSF negative samples were confirmed in serum (3 NS1 Ag and 7 positive IgM). Nine fatalities.	Asia, India	1–84 y	NS1, IgM ELISA	[119]
Four of 700 cases with viral infection with positive RT/PCR and IgM in CSF.	Brazil, Americas	<1–60 y	IgM ELISA, RT/PCR	[120]
A total of 1574 patients were included, 221 of whom developed central nervous system signs. These signs were spontaneously resolutive. There were no details regarding whether these incident signs were linked to encephalopathy or encephalitis.	French Guyana, Americas		NS1, IgM ELISA, RT/PCR	[121]

* Dengue fever (DF), Dengue Hemorrhagic Fever (DHF), Dengue Shock Syndrome (DSS).
